# Supplementary material for: Integrative brain transcriptome analysis links complement component 4 and HSPA2 to the APOE ε2 protective effect in Alzheimer disease
Source: Mol Psychiatry. 2021 Sep 3;26(10):6054–64. doi: 10.1038/s41380-021-01266-z (PMC8758485; doi:10.1038/s41380-021-01266-z)

SUPPLEMENTARY INFORMATION

Integrative Brain Omics Analysis Links Complement Pathway to the *APOE* ε2 Protective Effect in Alzheimer Disease

Rebecca Panitch, Junming Hu, Jaeyoon Chung, Congcong Zhu, Gaoyuan Meng, Weiming Xia, David A. Bennett, Kathryn L. Lunetta, Tsuneya Ikezu, Rhoda Au, Thor D. Stein, Lindsay A. Farrer, Gyungah R. Jun

Supplementary Table 1. Sample characteristics by source and *APOE* genotype

Dataset	APOE Genotype	AD Cases					Controls				
		N	% Female	AAD	PMI	RIN	N	% Female	AAD	PMI	RIN
ROSMAP	ε2/ε2	0	NA	NA	NA	NA	5	60.0	92.8 ± 4.5	6.4 ± 2.4	6.2 ± 0.9
	ε2/ε3	38	81.6	92.7 ± 6.7	7.8 ± 7.0	7.3 ± 1.0	44	65.9	88.4 ± 8.5	6.2 ± 3.8	7.2 ± 1.2
	ε3/ε3	212	65.6	90.2 ± 5.9	7.8 ± 5.3	7.0 ± 1.0	168	58.9	86.5 ± 6.9	7.2 ± 4.3	7.3 ± 1.0
	ε2/ε4	12	58.3	89.9 ± 3.4	6.8 ± 2.5	7.1 ± 1.0	4	50.0	89.0 ± 5.8	6.5 ± 5.5	6.9 ± 1.3
	ε3/ε4	114	65.8	88.7 ± 5.6	7.1 ± 4.4	7.0 ± 0.9	25	56.0	85.7 ± 7.8	7.3 ± 5.0	7.2 ± 1.1
	ε4/ε4	4	50.0	89.9 ± 1.3	13.0 ± 7.9	6.7 ± 0.5	1	100	85.4	5.8	5.9
	TOTAL	380	66.8	90.0 ± 5.9	7.6 ± 5.2	7.0 ± 0.9	247	59.9	86.9 ± 7.3	7.0 ± 4.2	7.2 ± 1.0
MAYO	ε2/2	0	NA	NA	NA	NA	0	NA	NA	NA	NA
	ε2/3	4	50.0	85.8 ± 6.6	NA	8.9 ± 0.2	12	41.7	81.4 ± 10.0	NA	7.7 ± 1.0
	ε3/3	35	54.3	83.7 ± 8.0	NA	8.6 ± 0.5	58	48.3	82.4 ± 9.1	NA	7.6 ± 1.1
	ε2/4	0	NA	NA	NA	NA	1	100	90	NA	6.7
	ε3/4	36	63.9	82.9 ± 6.8	NA	8.5 ± 0.6	8	50.0	84.1 ± 5.4	NA	7.6 ± 1.0
	ε4/4	7	71.4	74.2 ± 6.0	NA	8.5 ± 0.5	1	100	86.0	NA	5.9
	TOTAL	82	59.8	82.7 ± 7.6	NA	8.6 ± 0.5	80	48.8	82.6 ± 8.8	NA	7.6 ± 1.0
FHS/ BUADC	ε2/2	1	100	68.0	NA	4.5	2	50.0	77.5 ± 20.5	NA	7.3 ± 1.1
	ε2/3	6	66.7	84.8 ± 18.5	NA	5.8 ± 1.4	31	61.3	82.0 ± 11.2	NA	6.1 ± 1.4
	ε3/3	33	33.3	90.5 ± 8.0	NA	6.9 ± 1.3	76	43.4	87.2 ± 8.6	NA	6.8 ± 1.3
	ε2/4	3	66.7	80.7 ± 4.2	NA	5.5 ± 1.3	8	62.5	76.0 ± 11.0	NA	6.2 ± 1.1
	ε3/4	19	42.1	89.8 ± 6.4	NA	6.6 ± 1.2	12	58.3	82.8 ± 10.4	NA	7.0 ± 1.2
	ε4/4	2	50.0	79.0 ± 18.3	NA	6.7 ± 0.6	0	NA	NA	NA	NA
	TOTAL	64	42.2	88.6 ± 9.7	NA	6.6 ± 1.3	129	50.4	84.7 ± 10.2	NA	6.6 ± 1.3

N: sample size; AAD: Age at death; PMI: Post-mortem interval; RIN: RNA integrity number. NA: not available. Values for AAD, PMI and RIN expressed as mean ± standard deviation. A diagnosis of definite AD was established according to the National Institute of Aging (NIA) Reagan criteria.

Supplementary Table 3. Significantly differentially expressed genes ($P<10^{-6}$) in the total sample overlapping with genome-wide significant AD GWAS loci *

Gene	*APOE* ε2/ε3		*APOE* ε3/ε3		APOE ε3/ε4		Total	
	Z-score	P-value	Z-score	P-value	Z-score	P-value	Z-score	P-value
WDR81	1.50	0.13	3.80	$1.4x10^{-4}$	4.17	$3.0x10^{-5}$	5.74	$9.7x10^{-9}$
ICA1	0.03	0.98	-3.95	$7.7x10^{-5}$	-2.74	$6.2x10^{-3}$	-5.58	$2.5x10^{-8}$
TNIP1	1.03	0.31	3.06	$2.3x10^{-3}$	2.90	$3.8x10^{-3}$	5.02	$5.1x10^{-7}$
JAZF1	-0.64	0.52	-4.13	$3.7x10^{-5}$	-2.58	$9.8x10^{-3}$	-5.02	$5.1x10^{-7}$

* Bellenguez C, Küçükali F, Jansen I, Andrade V, Morenau-Grau S, Amin N, et al. Large meta-analysis of genome-wide association studies expands knowledge of the genetic etiology of Alzheimer’s disease and highlights potential translational opportunities. MedRxiv. 2020:2020.10.01.20200659.

Supplementary Table 4. Top-ranked differentially expressed genes in the *APOE* ε2/ε3 group

Gene	ROSMAP		MAYO		FHS/BUADC		Total	
	LogFC	P-value	LogFC	P-value	LogFC	P-value	Z-score	P-value
C4A	1.05	$6.0x10^{-5}$	1.42	0.05	0.30	0.66	4.28	$1.9x10^{-5}$
C4B	1.00	$7.3x10^{-5}$	0.98	0.12	0.30	0.67	4.10	$4.2x10^{-5}$
GFAP	0.89	$1.6x10^{-5}$	0.86	0.15	-0.12	0.81	4.12	$3.8x10^{-5}$
NPNT	0.40	0.02	1.03	$2.1x10^{-3}$	0.46	0.18	3.65	$2.6x10^{-4}$

Log2 fold change of expression levels (LogFC) between AD cases and controls was calculated within each dataset and the results were combined by meta-analysis.

Supplementary Table 5. Top-ranked differentially expressed genes (p<0.01) in each *APOE* genotype group in the combined ROSMAP, MAYO, and FHS/BUADC sample

Gene	***APOE* ε2/ε3**		***APOE* ε3/ε3**		**APOE ε3/ε4**		**Total**	
	**Z-score**	**P-value**	**Z-score**	**P-value**	**Z-score**	**P-value**	**Z-score**	**P-value**
C1QTNF5	3.18	$1.5x10^{-3}$	6.02	$1.8x10^{-9}$	4.08	$4.6x10^{-5}$	8.45	$2.8x10^{-17}$
SLC6A9	2.73	$6.3x10^{-3}$	5.18	$2.2x10^{-7}$	3.50	$4.7x10^{-4}$	8.22	$2.1x10^{-16}$
S100A4	2.65	$8.0x10^{-3}$	4.42	$9.9x10^{-6}$	3.54	$4.1x10^{-4}$	7.59	$3.3x10^{-14}$
ADAMTS2	2.65	$8.1x10^{-3}$	4.10	$4.1x10^{-5}$	4.20	$1.7x10^{-4}$	7.22	$5.1x10^{-13}$
MUC1	3.12	$1.8x10^{-3}$	3.94	$8.0x10^{-5}$	4.04	$5.3x10^{-5}$	7.02	$2.6x10^{-12}$
CHD4	2.89	$3.9x10^{-3}$	4.21	$2.6x10^{-5}$	4.69	$2.8x10^{-6}$	6.95	$3.7x10^{-12}$
SH3PXD2B	3.06	$2.2x10^{-3}$	3.70	$2.2x10^{-4}$	3.62	$2.9x10^{-4}$	6.40	$1.6x10^{-10}$
DUSP4	-2.96	$3.0x10^{-3}$	-3.05	$2.3x10^{-3}$	-2.69	$7.1x10^{-3}$	-5.82	$6.0x10^{-9}$
SLC25A26	-2.67	$7.5x10^{-3}$	-3.32	$9.0x10^{-4}$	-3.02	$2.6x10^{-3}$	-5.77	$8.1x10^{-9}$
RRNAD1	2.81	$4.9x10^{-3}$	3.03	$2.5x10^{-3}$	2.70	$6.9x10^{-3}$	5.44	$5.3x10^{-8}$
RIMS1	-2.82	$4.8x10^{-3}$	-3.02	$2.5x10^{-3}$	-3.27	$1.1x10^{-3}$	-5.17	$2.3x10^{-7}$

Results were generated from bulk RNA sequence data and combined across data sets by meta-analysis of differential expression between AD and control brains.

Supplementary Table 6. Distribution of neurofibrillary tangles (quantified by Braak Stage) and neuritic plaques (quantified by CERAD plaque score) by dataset and *APOE* genotype

Dataset	Subgroup	Braak Stage		Plaque Score	
		N	Mean+SD	N	Mean+SD
ROSMAP	*APOE* ε2/ε3	71	-0.43 ± 0.8	71	0.55 ± 0.9
	APOE ε3/ε3	349	-0.20 ± 1.0	349	0.18 ± 1.0
	APOE ε3/ε4	125	0.36 ± 0.9	125	-0.45 ± 0.9
	Total	568	-0.10 ± 1.0	568	0.08 ± 1.0
FHS/BUDADC	*APOE* ε2/ε3	15	-0.55 ± 0.7	13	0.52 ± 0.8
	APOE ε3/ε3	99	-0.09 ± 1.0	77	0.13 ± 1.0
	APOE ε3/ε4	30	0.47 ± 1.0	26	-0.45 ± 0.9
	Total	151	-0.03 ± 1.0	122	0.06 ± 1.0

N= sample size; SD= standard deviation

Plaque (CERAD) scores reflect the density of neuritic plaques: 1 (definite), 2 (probable), 3 (possible, and 4 (none); Braak stages represent the spatial distribution of neurofibrillary tangles: 0 (none), I-II (transentorhinal and entorhinal cortex), III-IV (hippocampal and neighboring limbic areas), and V-VI (neocortical areas). All traits were rank-transformed for age of death and sex.

Supplementary Table 7. Association of expression of top-ranked differentially expressed genes among *APOE* ε2/ε3 subjects with neurofibrillary tangles (Braak stage) and neuritic plaques (plaque score) in brains from AD cases and controls in the ROSMAP and FHS/BUADC datasets.

**Gene**	**Braak Stage**						**Plaque Score**					
	**ROSMAP**		**FHS/BUADC**		**Meta-Analysis**		**ROSMAP**		**FHS/BUADC**		**Meta-Analysis**	
	**β**	**P-value**	**β**	**P-value**	**β**	**P-value**	**β**	**P-value**	**β**	**P-value**	**β**	**P-value**
C4A	0.15	5.4×10^{-4}	0.09	0.36	0.14	3.7×10^{-4}	-0.13	2.8×10^{-3}	-0.14	0.23	-0.13	1.2×10^{-3}
C4B	0.16	2.6×10^{-4}	0.13	0.21	0.16	1.1×10^{-4}	-0.16	5.3×10^{-4}	-0.19	0.11	-0.16	1.3×10^{-4}
GFAP	0.21	5.4×10^{-6}	0.08	0.30	0.18	9.1×10^{-6}	-0.18	1.8×10^{-4}	-0.13	0.13	-0.17	5.5×10^{-5}
NPNT	0.32	1.4×10^{-6}	0.09	0.11	0.19	1.2×10^{-5}	-0.27	7.4×10^{-5}	-0.17	6.9×10^{-3}	-0.22	2.2×10^{-6}

Expression differences (β estimate represent the magnitude of effect) for each trait were calculated using a linear regression model separately in each dataset and the results were combined by meta-analysis.

Supplementary Table 8. Association of expression of top-ranked differentially expressed genes among *APOE* ε2/ε3 subjects with neurofibrillary tangles (Braak stage) and neuritic plaques (plaque score) in the ROSMAP dataset

**Gene**	***APOE* genotype**	**Braak Stage**		**Plaque Score**	
		**β**	**P-value**	**β**	**P-value**
C4A	ε2/ε3	0.22	9.8×10^{-3}	-0.27	5.2×10^{-3}
	ε3/ε3	0.15	8.9×10^{-3}	-0.05	0.35
	ε3/ε4	0.02	0.85	-0.16	0.10
	Total	0.15	5.4×10^{-4}	-0.13	2.8×10^{-3}
C4B	ε2/ε3	0.20	0.03	-0.31	2.2×10^{-3}
	ε3/ε3	0.17	3.3×10^{-3}	-0.08	0.13
	ε3/ε4	0.05	0.63	-0.18	0.08
	Total	0.16	2.6×10^{-4}	-0.16	5.3×10^{-4}
GFAP	ε2/ε3	0.26	7.1×10^{-3}	-0.33	2.5×10^{-3}
	ε3/ε3	0.18	3.4×10^{-3}	-0.09	0.15
	ε3/ε4	0.12	0.22	-0.09	0.34
	Total	0.21	5.4×10^{-6}	-0.18	1.8×10^{-4}
NPNT	ε2/ε3	0.16	0.29	-0.25	0.14
	ε3/ε3	0.38	5.9×10^{-6}	-0.28	5.7×10^{-4}
	ε3/ε4	0.12	0.43	0.04	0.79
	Total	0.32	1.4×10^{-6}	-0.27	7.4×10^{-5}

Expression differences (β estimate represent the magnitude of effect) for each trait were calculated using a linear regression model separately in each dataset and the results were combined by meta-analysis.

Supplementary Table 9. Robust gene co-expression networks derived from the ROSMAP sample using various sources of AD-associated seed genes that are preserved in MAYO and/or FHS/BUADC datasets

Mod	Total Genes	*APOE* and AD Status Subgroup	Preservation Score[1]		Source of Genes Used to Seed Co-expression Networks					
					DEGs in Total Sample[2]		DEGs in *APOE* Subgroup[3]		AD GWAS[4]	
			MAYO	FHS/BUADC	# Genes	ENR-P	# Genes	ENR-P	# Genes	ENR-P
M01	674	23ad	28.2	-0.8	96	$4.3x10^{-32}$	8	0.05	66	$1.2x10^{-5}$
M02	1501	33ad	28.7	25.1	108	$1.2x10^{-16}$	430	$7.1x10^{-86}$	111	$8.4x10^{-6}$
M03	112	33ad	30.7	5.7	23	$7.0x10^{-12}$	56	$1.1x10^{-23}$	18	$1.6x10^{-4}$
M04	203	33ad	35.8	7.0	71	$3.5x10^{-55}$	121	$3.3x10^{-63}$	21	0.02
M05	449	33ad	58.9	31.7	39	$7.3x10^{-8}$	222	$1.8x10^{-94}$	45	$2.8x10^{-5}$
M06	495	33ad	61.2	23.4	35	$7.6x10^{-5}$	90	$1.6x10^{-5}$	47	$7.2x10^{-5}$
M07	774	33ad	120.6	37.5	126	$7.6x10^{-56}$	472	$8.1x10^{-260}$	73	$1.1x10^{-7}$
M08	247	33ad	16.9	6.4	59	$7.0x10^{-4}$	59	$1.1x10^{-7}$	24	0.02
M09	546	33ad	205.9	39.0	84	$8.2x10^{-35}$	379	$3.0x10^{-238}$	78	$6.7x10^{-18}$
M10	2105	34ad	91.2	46.3	247	$5.9x10^{-77}$	350	$3.6x10^{-108}$	210	$6.8x10^{-24}$
M11	1242	34ad	14.3	23.7	69	$1.3x10^{-4}$	102	$1.4x10^{-7}$	99	$1.8x10^{-5}$
M12	407	34ad	42.6	17.6	152	$1.5x10^{-121}$	131	$6.9x10^{-74}$	45	$1.0x10^{-5}$
M13	491	34ad	86.0	39.0	76	$9.4x10^{-30}$	191	$1.1x10^{-126}$	51	$1.1x10^{-5}$
M14	314	33c	94.6	31.5	71	$1.0x10^{-39}$	236	$9.5x10^{-157}$	32	$1.7x10^{-3}$
M15	707	33c	16.4	28.1	44	$2.7x10^{-4}$	161	$6.2x10^{-18}$	59	$5.7x10^{-4}$
M16	779	33c	166.7	51.4	128	$1.4x10^{-55}$	521	$1.3x10^{-310}$	108	$6.2x10^{-23}$
M17	198	33c	23.3	7.6	32	$4.1x10^{-13}$	84	$4.4x10^{-28}$	27	$3.1x10^{-5}$
M18	910	33c	30.6	39.5	72	$4.0x10^{-12}$	335	$2.0x10^{-94}$	80	$1.7x10^{-6}$
M19	599	33c	41.4	24.8	70	$9,6x10^{-21}$	148	$6.1x10^{-20}$	59	$2.5x10^{-6}$
M20	2234	33c	43.9	45.0	103	$4.0x10^{-4}$	512	$3.7x10^{-61}$	158	$2.5x10^{-6}$
M21	320	34c	51.4	19.0	50	$1.1x10^{-17}$	136	$1.9x10^{-89}$	38	$9.1x10^{-5}$
M22	254	34c	34.3	19.4	37	$5.2x10^{-12}$	35	$2.4x10^{-06}$	38	$2.0x10^{-7}$
M23	782	34c	90.1	36.0	144	$3.3x10^{-62}$	162	$1.1x10^{-54}$	91	$2.0x10^{-11}$

ENR-P: Bonferroni corrected enrichment P-value; DEGs: differentially expressed genes.

[1] Preservation score: Z-summary statistic representing a network identified in the ROSMAP dataset across the MAYO and FHS/BUADC datasets. Interpretation of Z-summary statistic: < 2 = no preservation, 2-10 = moderate preservation, > 10 = high preservation.

[2] Includes DEGs in the total sample with $p<10^{-6}$

[3] Includes moderately significant DEGs in the corresponding *APOE* subgroup ($p<0.01$) that were also moderately significant in the total sample ($p<0.01$)

[4] Includes genes with AD association p-value $<10^{-3}$ large GWAS (Kunkle et al. Nature Genetics 2019; 51:414-430)

Supplementary Table 10. Enrichment of highly preserved co-expression networks for brain cell type genes in the ROSMAP sample

Module	Total Genes	*APOE* and AD Status Subgroup	Enrichment P-value					
			Ast	Mic	Oli	OPC	In Neu	Ex Neu
M01	674	23ad	$5.2x10^{-59}$	NS	$9.1x10^{-57}$	$6.8x10^{-44}$	NS	NS
M02	1501	33ad	NS	NS	NS	0.02	0.02	NS
M03	112	33ad	$8.4x10^{-3}$	$5.5x10^{-6}$	$1.9x10^{-43}$	$3.2x10^{-6}$	NS	NS
M04	203	33ad	$1.1x10^{-6}$	NS	$2.8x10^{-5}$	$8.7x10^{-9}$	NS	NS
M05	449	33ad	NS	$1.6x10^{-7}$	NS	$5.3x10^{-10}$	$1.1x10^{-4}$	$2.8x10^{-18}$
M06	495	33ad	$2.6x10^{-97}$	0.01	$1.1x10^{-12}$	$2.4x10^{-44}$	NS	NS
M07	774	33ad	NS	0.02	NS	$1.2x10^{-5}$	$5.2x10^{-14}$	$7.6x10^{-147}$
M08	247	33ad	NS	$8.1x10^{-5}$	$1.7x10^{-6}$	NS	NS	NS
M09	546	33ad	NS	NS	NS	$3.6x10^{-6}$	$1.3x10^{-41}$	$1.6x10^{-313}$
M10	2105	34ad	NS	$1.0x10^{-3}$	NS	$6.1x10^{-14}$	$3.3x10^{-41}$	$2.6x10^{-277}$
M11	1242	34ad	NS	NS	NS	$6.9x10^{-3}$	NS	NS
M12	407	34ad	$1.0x10^{-22}$	$4.9x10^{-3}$	$5.0x10^{-8}$	$5.6x10^{-20}$	NS	NS
M13	491	34ad	NS	$1.7x10^{-3}$	$5.3x10^{-3}$	$3.5x10^{-12}$	$1.9x10^{-7}$	$2.6x10^{-66}$
M14	314	33c	NS	NS	NS	0.02	$3.9x10^{-9}$	$7.4x10^{-96}$
M15	707	33c	NS	NS	NS	$5.0x10^{-3}$	NS	NS
M16	779	33c	NS	NS	NS	$5.7x10^{-7}$	$4.5x10^{-40}$	0
M17	198	33c	$6.9x10^{-5}$	$7.4x10^{-11}$	$1.4x10^{-29}$	$1.1x10^{-9}$	0.03	NS
M18	910	33c	NS	NS	NS	NS	$4.7x10^{-8}$	$2.2x10^{-5}$
M19	599	33c	$3.2x10^{-118}$	$4.5x10^{-4}$	$8.0x10^{-16}$	$3.1x10^{-32}$	NS	NS
M20	2234	33c	NS	NS	NS	NS	$6.6x10^{-10}$	$6.8x10^{-64}$
M21	320	34c	NS	$3.6x10^{-6}$	NS	$1.7x10^{-8}$	$2.3x10^{-3}$	$5.5x10^{-29}$
M22	254	34c	NS	NS	NS	NS	NS	$9.1x10^{-38}$
M23	782	34c	NS	NS	NS	$2.9x10^{-6}$	$3.1x10^{-38}$	$2.8x10^{-277}$

NS: not significant; Ast: astrocytes; Ex Neu: excitatory neurons; In Neu: inhibitory neurons; Mic: microglia; Oli: oligodendrocytes; OPC: oligodendrocyte progenitor cells

Supplementary Table 11. Association of Expression of Network M01 Genes with Braak Stage and Plaque Score

Gene	Braak Stage						Plaque Score					
	ROSMAP		FHS/BUADC		Meta-Analysis		ROSMAP		FHS/BUADC		Meta-Analysis	
	β	P-value	β	P-value	β	P-value	β	P-value	β	P-value	β	P-value
ARHGEF37	0.23	6.3×10^{-3}	0.15	8.0×10^{-3}	0.17	1.7×10^{-4}	-0.29	7.2×10^{-4}	-0.1	0.12	-0.17	1.0×10^{-3}
C4A	0.15	5.4×10^{-4}	0.09	0.36	0.14	3.7×10^{-4}	-0.13	2.8×10^{-3}	-0.14	0.23	-0.13	1.2×10^{-3}
C4B	0.16	2.6×10^{-4}	0.13	0.21	0.16	1.1×10^{-4}	-0.16	5.3×10^{-4}	-0.19	0.11	-0.16	1.3×10^{-4}
C5orf64	0.08	0.17	0.1	0.15	0.09	0.05	-0.11	0.09	-0.07	0.31	-0.09	0.05
CALD1	0.18	0.06	0.09	0.02	0.1	4.2×10^{-3}	-0.22	0.02	-0.07	0.09	-0.1	0.01
CHST6	0.13	0.02	0.16	0.03	0.14	1.6×10^{-3}	-0.16	5.3×10^{-3}	-0.16	0.05	-0.16	6.2×10^{-4}
DOCK1	0.17	0.05	0.06	0.12	0.08	0.02	-0.23	6.6×10^{-3}	-0.04	0.38	-0.08	0.04
DOCK5	0.19	0.01	0.1	0.15	0.14	5.9×10^{-3}	-0.23	2.8×10^{-3}	-0.05	0.55	-0.14	0.01
HIP1	0.12	0.16	0.14	6.9×10^{-3}	0.13	2.1×10^{-3}	-0.25	3.4×10^{-3}	-0.11	0.06	-0.15	1.3×10^{-3}
HSPA2	0.2	1.6×10^{-3}	0.14	0.09	0.18	3.9×10^{-4}	-0.33	4.7×10^{-7}	-0.10	0.27	-0.25	1.6×10^{-6}
LPAR1	0.12	0.06	0.15	0.02	0.14	2.1×10^{-3}	-0.23	3.7×10^{-4}	-0.09	0.19	-0.17	4.5×10^{-4}
MCM7	0.24	0.01	0.14	3.4×10^{-4}	0.15	1.4×10^{-5}	-0.35	3.1×10^{-4}	-0.11	0.01	-0.15	1.3×10^{-4}
PHLPP1	0.24	0.02	0.07	0.2	0.1	0.03	-0.38	3.8×10^{-4}	-0.04	0.5	-0.11	0.02
PLEKHA7	0.09	0.27	0.13	0.03	0.12	0.01	-0.18	0.03	-0.09	0.19	-0.12	0.02
PRIMA1	0.15	0.03	0.13	0.15	0.14	8.8×10^{-3}	-0.22	1.5×10^{-3}	-0.09	0.35	-0.18	1.6×10^{-3}
RHOU	0.21	9.2×10^{-3}	0.04	0.49	0.1	0.04	-0.22	6.8×10^{-3}	-0.04	0.56	-0.11	0.03
RNF43	-0.23	3.1×10^{-3}	-0.07	0.3	-0.13	6.6×10^{-3}	0.19	0.02	0.06	0.42	0.11	0.03
SLC45A3	0.17	0.01	0.13	0.16	0.16	4.0×10^{-3}	-0.29	1.9×10^{-5}	-0.08	0.44	-0.23	4.7×10^{-5}
TNS3	0.14	0.04	0.09	0.1	0.11	8.7×10^{-3}	-0.18	6.2×10^{-3}	-0.04	0.46	-0.11	0.02

Expression of genes listed were nominally associated ($P<0.05$) in both traits in the meta-analysis. Expression differences (β estimate represent the magnitude of effect) for each trait were calculated using a linear regression model separately in each dataset and the results were combined by meta-analysis.

Supplementary Table 12. Differential expression of genes from the M01 network between AD cases and controls by brain cell type *

Gene	Mean Expression			Ast		Oli		OPC	
	**Ast**	**Oli**	**OPC**	**LogFC[1]**	**P-value**	**LogFC**	**P-value**	**LogFC**	**P-value**
CALD1	3.60	9.72	10.37	0.015	2.7E-01	-0.039	6.8E-01	-0.185	7.8E-03
DOCK1	NA	2.07	NA	NA	NA	0.245	**3.8E-07**	NA	NA
DOCK5	NA	10.99	5.07	NA	NA	0.075	1.8E-05	0.039	8.0E-01
HIP1	NA	5.06	9.13	NA	NA	0.026	9.3E-03	-0.013	9.0E-01
HSPA2	2.34	11.78	NA	-0.041	3.9E-01	0.139	**1.8E-10**	NA	NA
LPAR1	NA	4.55	NA	NA	NA	0.134	**7.0E-08**	NA	NA
PHLPP1	2.48	5.59	3.19	0.054	8.1E-01	0.185	**3.8E-11**	0.170	4.5E-01
RHOU	NA	7.04	2.90	NA	NA	0.096	3.9E-05	0.125	3.3E-01
SLC45A3	NA	2.14	NA	NA	NA	0.041	1.5E-02	NA	NA
TNS3	2.59	NA	NA	0.014	8.0E-01	NA	NA	NA	NA

* Genes with low expression (mean < 2) in oligodendrocytes, oligodendrocyte progenitor cells, and astrocytes were excluded.

[1] Log2 fold change of expression levels (LogFC) and P value (P) were calculated between AD and control brains from single nuclei RNA sequencing data from ROSMAP.

Ast: astrocyte; Oli: oligodendrocytes; OPC: oligodendrocyte progenitor cells; NA: not available due to small number of single cells showing expression (average expression < 2).

P values significant after false discovery rate (FDR) adjustment are highlighted in bold.

Supplementary Table 13. Characteristics of Immunoassay Measures in FHS/BUADC

Group	Aβ42		pTau181/tTau		pTau231/tTau		C4A		C4B		PSD95	
	N	Mean	N	Mean	N	Mean	N	Mean	N	Mean	N	Mean
Total	191	8.7×10^{-3}	146	-7.5×10^{-3}	186	0.04	179	-4.3×10^{-3}	179	-0.02	192	4.2×10^{-3}
ε2/ε3	37	-0.47	29	-0.02	37	-0.23	34	-0.21	34	-0.07	37	0.26
ε3/ε3	109	0.03	81	0.02	104	0.05	101	7.6×10^{-3}	101	0.03	109	2.3×10^{-3}
ε3/ε4	31	0.38	25	-0.05	31	0.30	30	0.23	30	-0.16	31	-0.25

N: sample size; SD: standard deviation; $A\beta_{42}$: amyloid-β 42; pTau181/tTau: ratio of phosphorylated tau at position 181 to total tau; pTau231/tTau: ratio of phosphorylated tau at position 231 to total tau; C4A: complement 4A protein; C4B: complement 4B protein; PSD95: postsynaptic density of 95k Dalton. All traits were rank-transformed for age of death and sex.

Supplementary Table 14. Association of Expression of *APOE* ε2/ε3-related Genes with Measures of AD-related Proteins

Gene *	Aβ42/Aβ40		pTau181/tTau		pTau231/tTau		PSD95		C4A		C4B	
	β	P	β	P	β	P	β	P	β	P	β	P
C4A	-0.06	0.26	$-9.1x10^{-3}$	0.89	0.22	$5.1x10^{-5}$	-0.10	0.08	0.06	0.26	0.06	0.33
C4B	-0.07	0.22	$-7.4x10^{-3}$	0.91	0.21	$7.0x10^{-5}$	-0.10	0.08	0.04	0.44	0.05	0.43
GFAP	-0.12	0.12	$-5.6x10^{-3}$	0.95	0.23	$1.5x10^{-3}$	-0.12	0.13	-0.05	0.54	0.08	0.31
NPNT	0.02	0.84	0.07	0.55	0.35	$4.9x10^{-4}$	-0.08	0.48	0.15	0.16	-0.14	0.23
DOCK1	-0.11	0.46	$-1.4x10^{-3}$	0.99	0.28	0.04	-0.20	0.18	-0.10	0.46	0.39	$8.3x10^{-3}$
HSPA2	-0.16	0.02	-0.01	0.86	0.30	$4.3x10^{-6}$	-0.10	0.17	0.10	0.13	0.20	$6.1x10^{-3}$
LPAR1	-0.11	0.26	-0.02	0.88	-0.05	0.60	$-6.1x10^{-3}$	0.95	-0.08	0.39	0.18	0.07
PHLPP1	-0.24	0.04	-0.10	0.44	0.38	$3.9x10^{-4}$	-0.18	0.13	0.16	0.15	0.21	0.08

* Includes significantly differentially expressed genes shown in Table S1 and Supplemental Table S11.

Aβ42/Aβ40: ratio of amyloid-β_{42} to amyloid-β_{40}; pTau181/tTau: ratio of phosphorylated tau at position 181 to total tau; pTau231/tTau: ratio of phosphorylated tau at position 231 to total tau; C4A: complement 4A protein; C4B: complement 4B protein; PSD95: postsynaptic density of 95k Dalton.

Supplementary Figure 1. Quality control of FHS/BUADC RNA sequencing data including the distribution among samples of (A) library size and (B) expression counts across batches. (C) First 2 principle components of gene expression for samples colored by (C) library batches and (D) sequencing batches.

A.

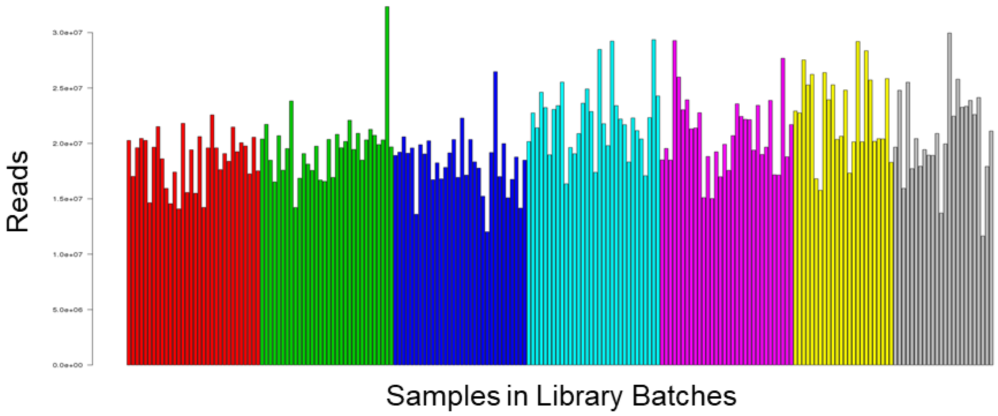

B.

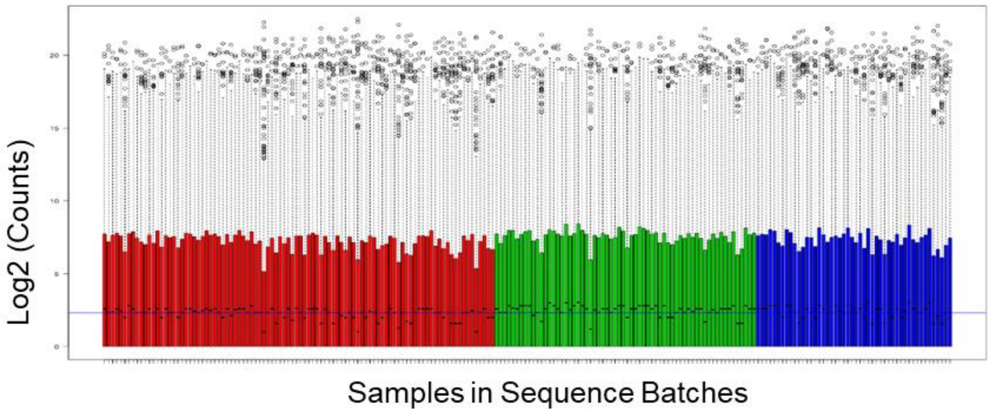

C.

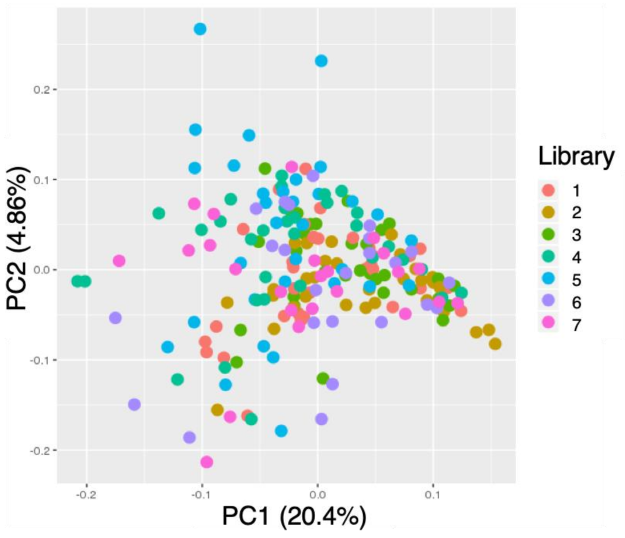

D.

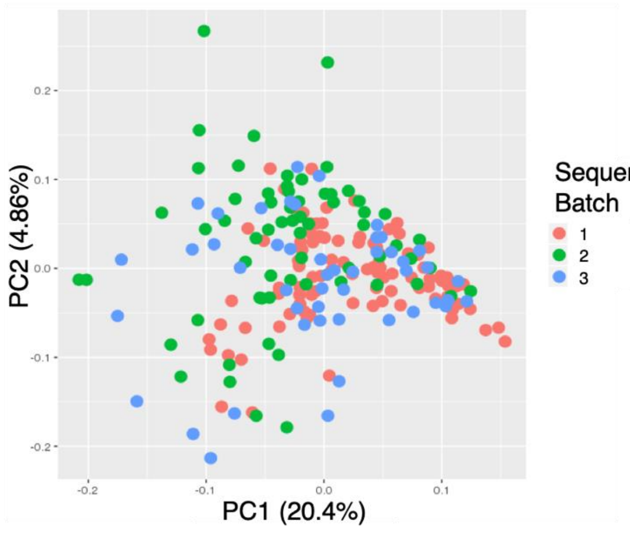

Supplementary Figure 2. Volcano Plots for Differential Gene Expression Analysis in the ROSMAP Sample Among Subjects with *APOE* Genotypes ε2/ε3 (panel A), ε3/ε3 (panel B) and ε3/ε4 (panel C), and in the Total Sample (panel D). Measure of differential expression between AD cases and controls expressed as Log_2 fold change is shown on the x-axis and significance level expressed as the -log P-value is shown on the y-axis. Results for each gene are represented by colored dots and are restricted to those with | log_2 fold-change | > 0.3 or p-value < 0.01. Genes that were surpassed the p-value and log_2 fold-change thresholds are shown in red, genes that surpassed the p-value threshold only are shown in blue, genes that surpassed the log_2 fold-change threshold only are shown in green, and genes that did not surpass either threshold are shown in grey.

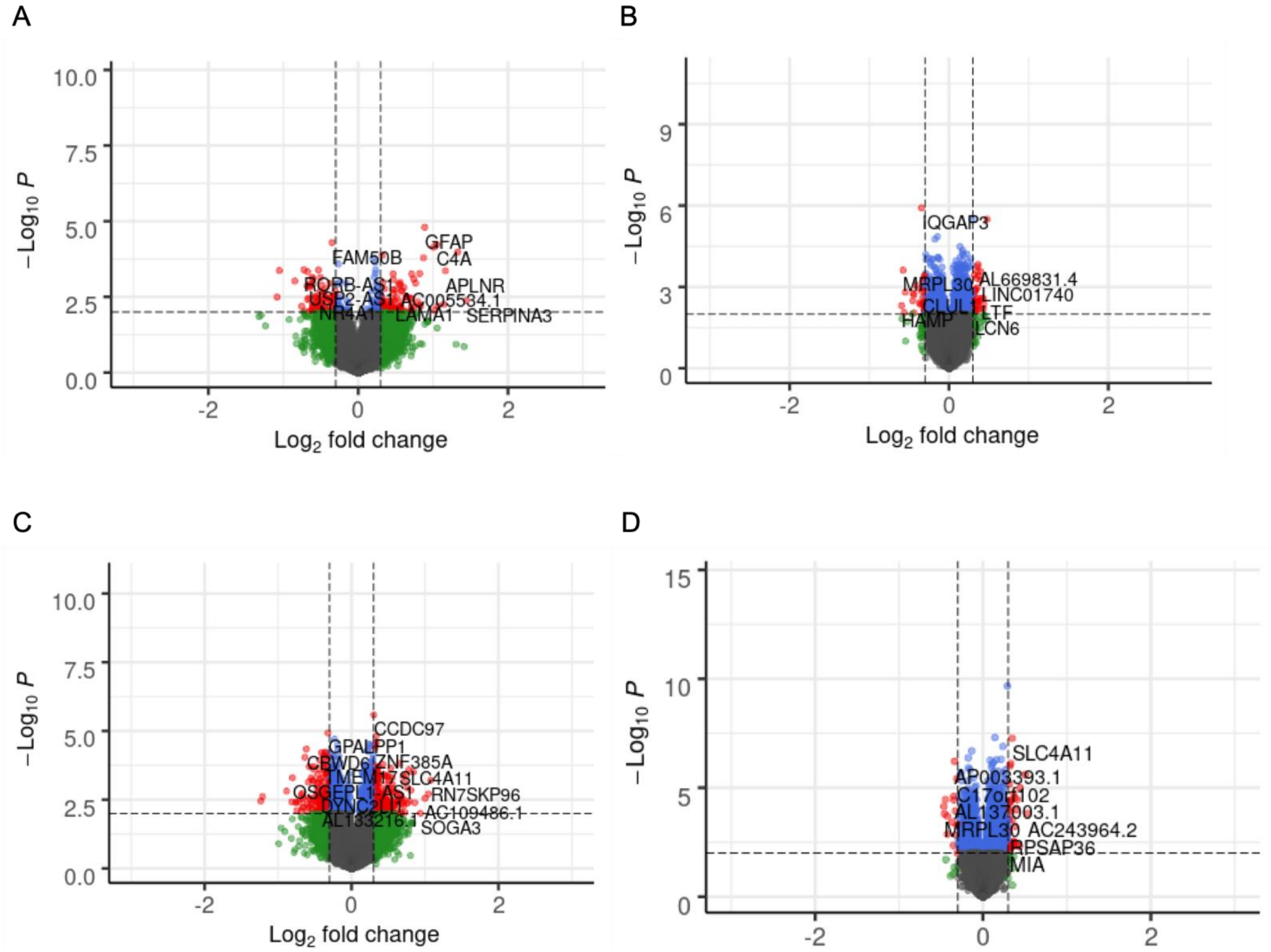

Supplementary Figure 3. Volcano Plots for Differential Gene Expression Analysis in the MAYO Sample Among Subjects with *APOE* Genotypes ε2/ε3 (panel A), ε3/ε3 (panel B) and ε3/ε4 (panel C), and in the Total Sample (panel D). Measure of differential expression between AD cases and controls expressed as Log_2 fold change is shown on the x-axis and significance level expressed as the -log P-value is shown on the y-axis. Results for each gene are represented by colored dots and are restricted to those with | log_2 fold-change | > 0.3 or p-value < 0.01. Genes that were surpassed the p-value and log_2 fold-change thresholds are shown in red, genes that surpassed the p-value threshold only are shown in blue, genes that surpassed the log_2 fold-change threshold only are shown in green, and genes that did not surpass either threshold are shown in grey.

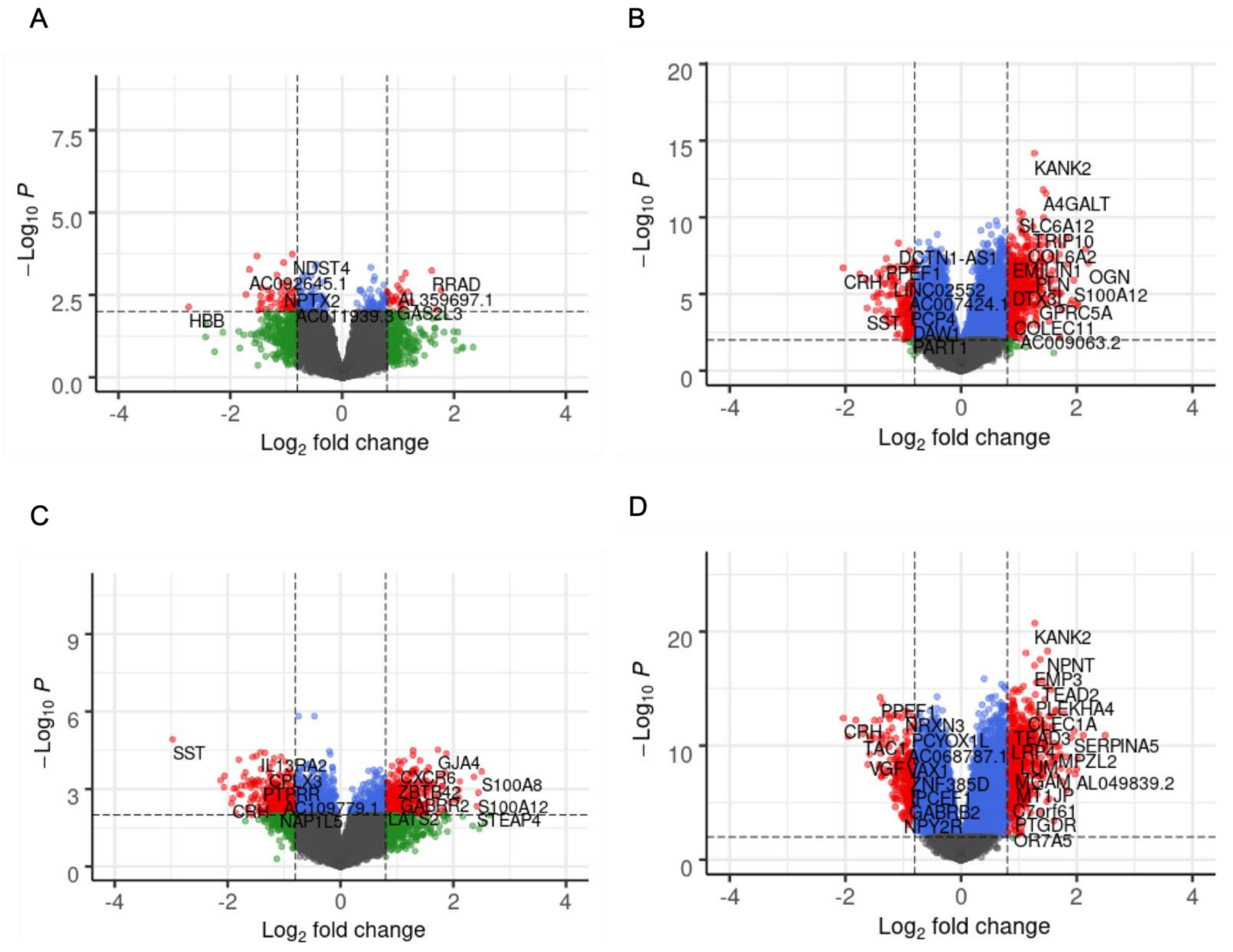

Supplementary Figure 4. Volcano Plots for Differential Gene Expression Analysis in the FHS/BUADC Sample Among Subjects with *APOE* Genotypes ε2/ε3 (panel A), ε3/ε3 (panel B) and ε3/ε4 (panel C), and in the Total Sample (panel D). Measure of differential expression between AD cases and controls expressed as Log_2 fold change is shown on the x-axis and significance level expressed as the -log P-value is shown on the y-axis. Results for each gene are represented by colored dots and are restricted to those with | $\log_2$ fold-change | > 0.3 or p-value < 0.01. Genes that were surpassed the p-value and $\log_2$ fold-change thresholds are shown in red, genes that surpassed the p-value threshold only are shown in blue, genes that surpassed the $\log_2$ fold-change threshold only are shown in green, and genes that did not surpass either threshold are shown in grey.

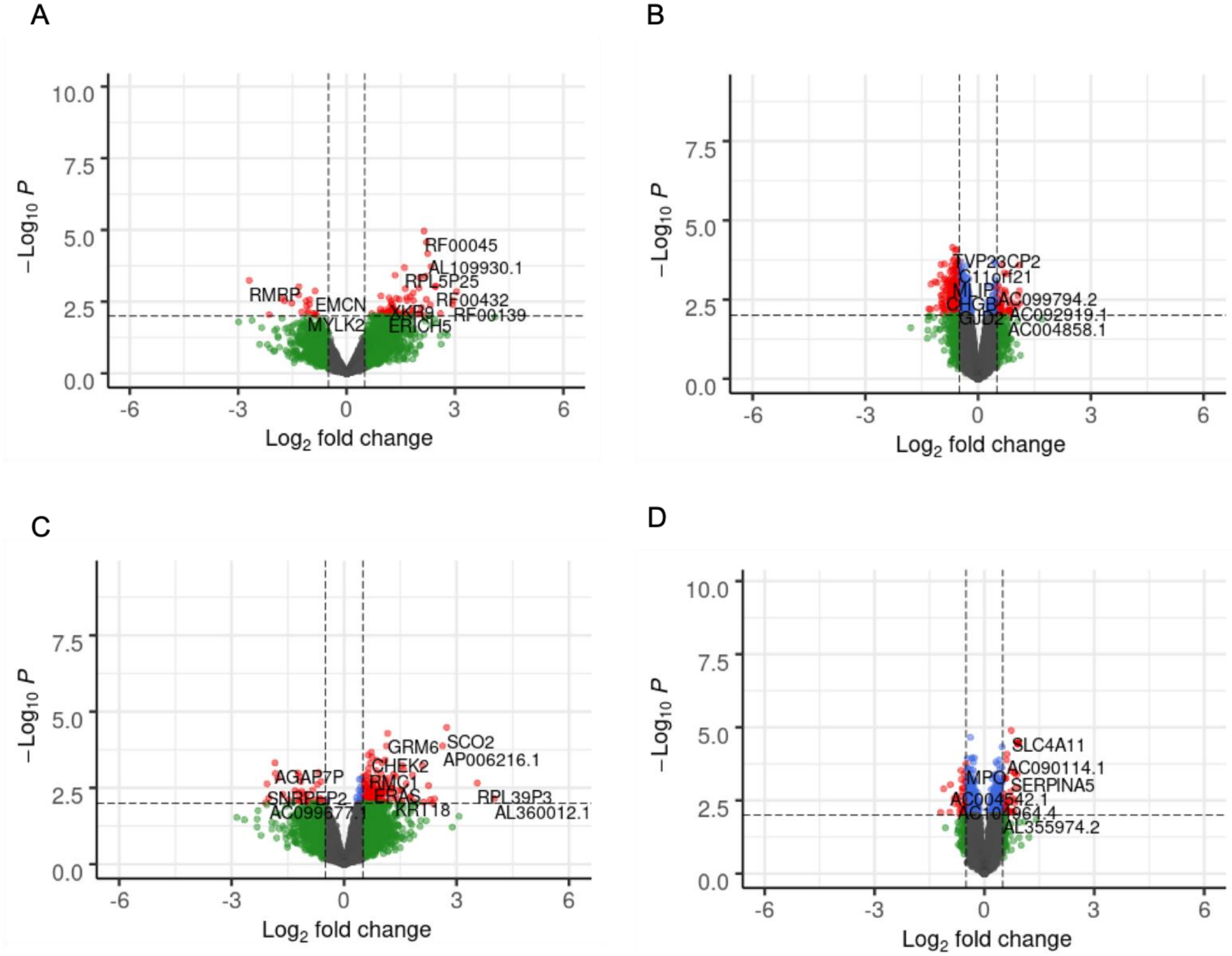

Supplementary Figure 5. Boxplots showing comparative expression across *APOE* genotype groups in ROSMAP Study controls for (A) *C4A* and (B) *C4B*. Pairwise P-values were calculated using wilcoxin rank sum tests.

(A)

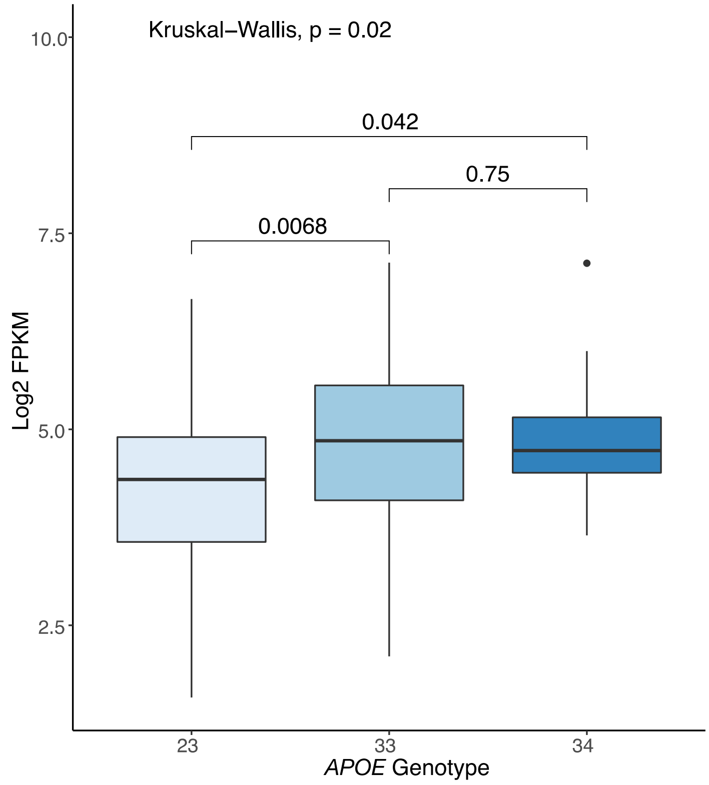

(B)

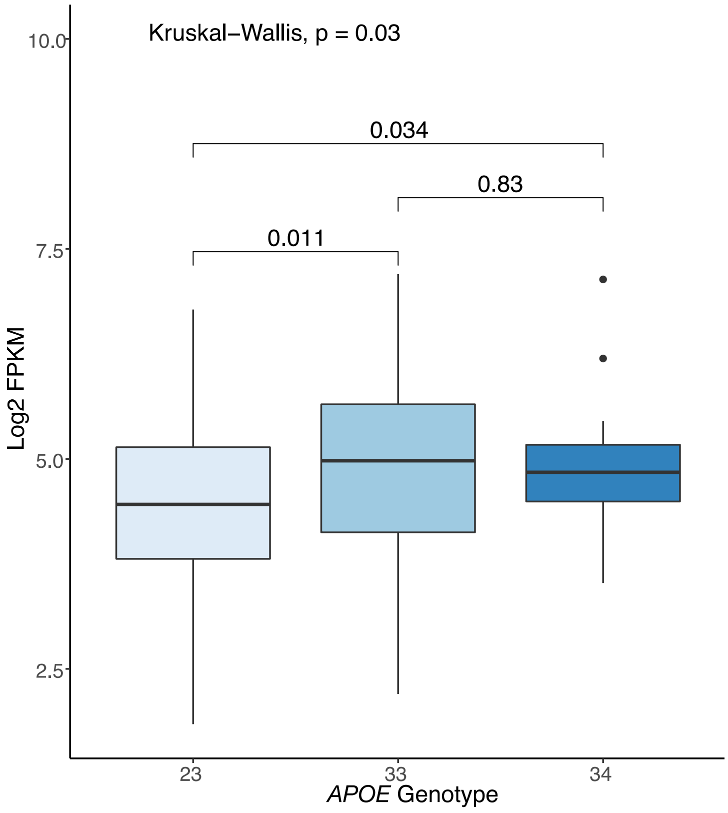

Supplementary Figure 6. Number of significant differentially expressed genes ($p<0.01$) in each of and across the *APOE* genotype groups (ε2/ε3, ε3/ε3, ε3/ε4) and the total sample.

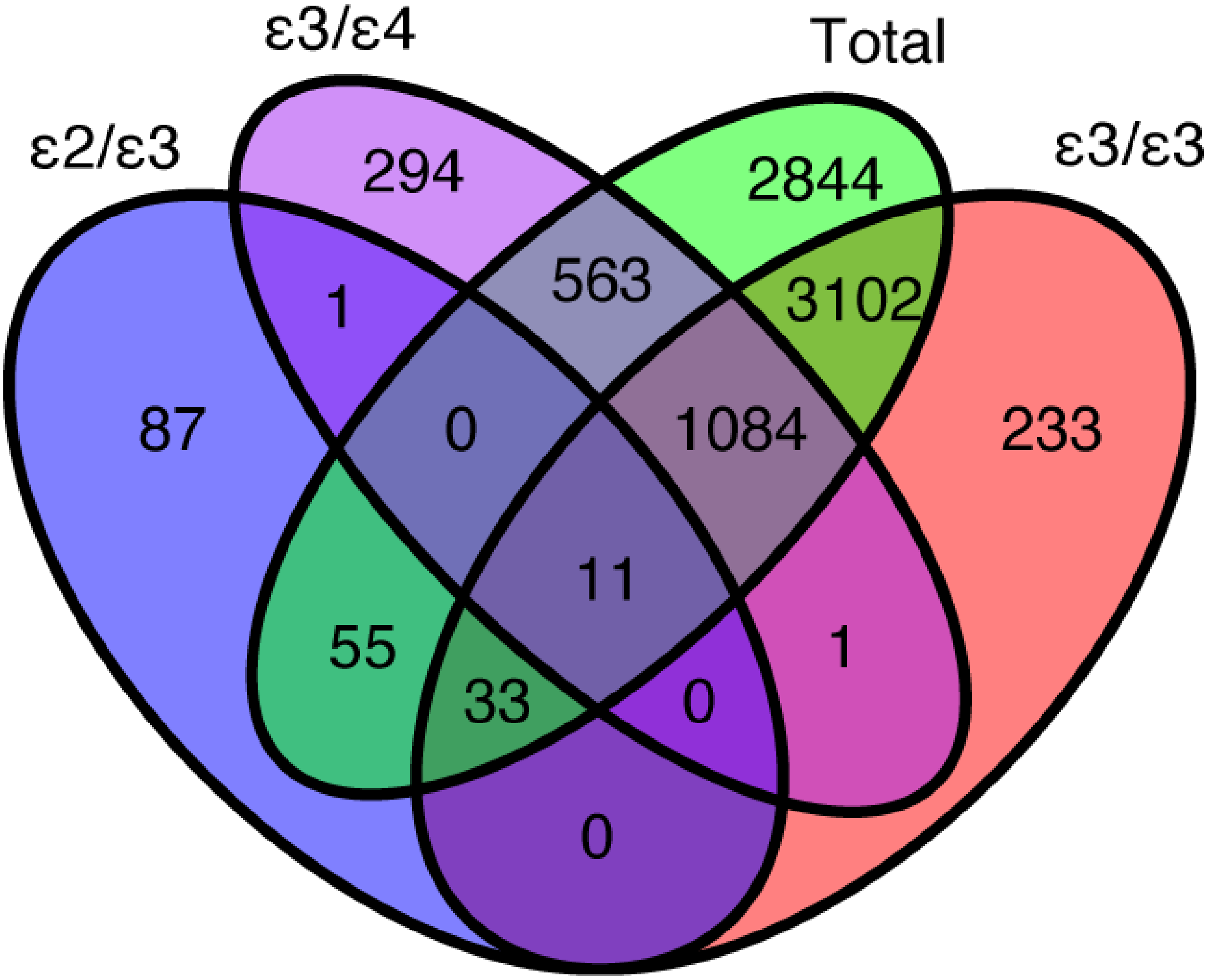

Supplementary Figure 7. Cell-type characterization of brain tissue obtained from 24 AD cases and 24 controls in the ROSMAP Study. Plot shows a two-dimensional t-distributed stochastic neighbor embedding (t-SNE) projection of all annotated cells derived from single nuclei RNA sequencing (snRNA-seq) data.

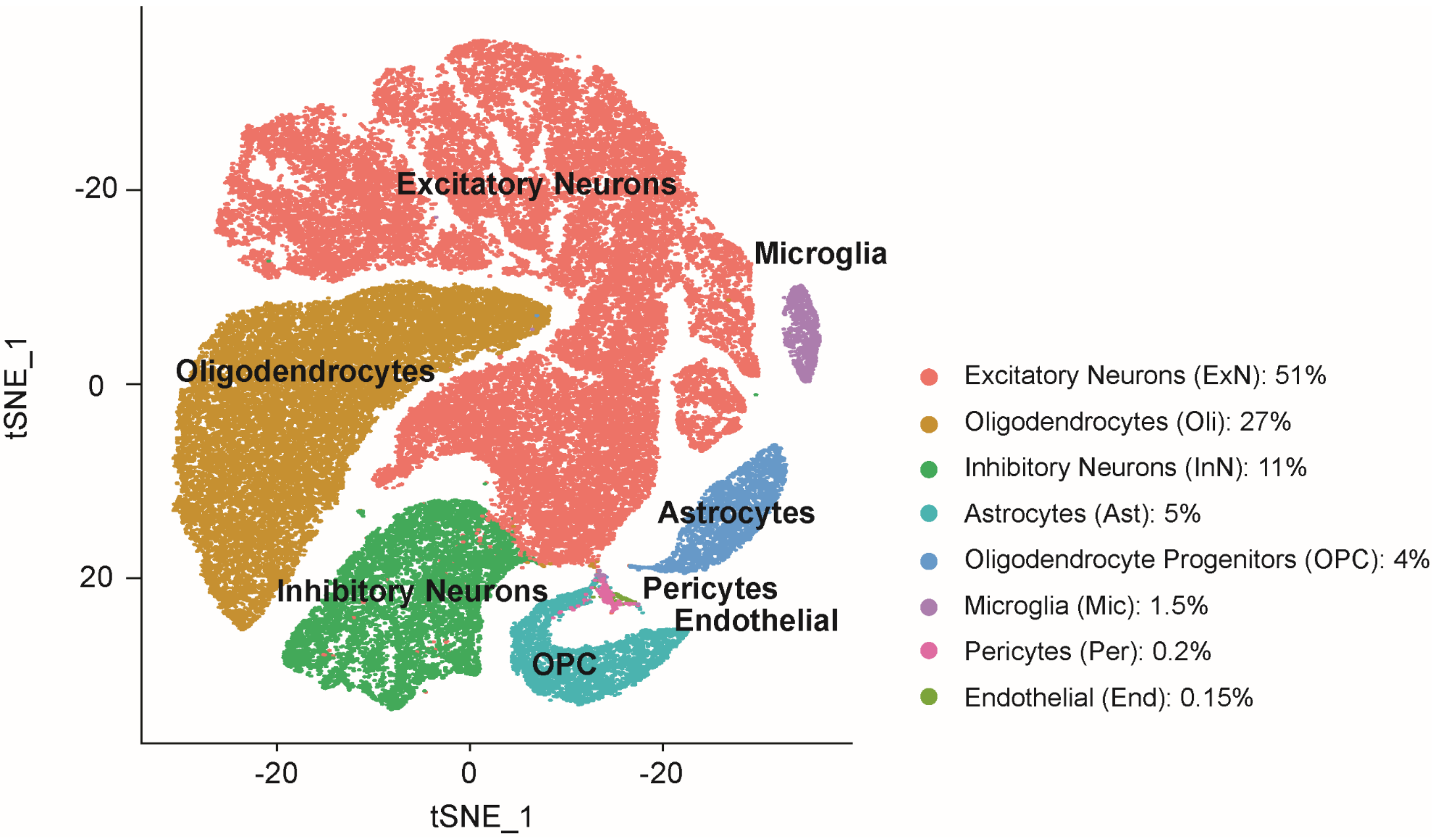

Supplementary Figure 8. Cell type-specific marker genes. (A) *SYT1* for neurons, (B) *GAD1* for excitatory neurons, (C) *SLC17A7* for inhibitory neurons, (D) *PLP1* for oligodendrocytes, (E) *AQP4* for oligodendrocyte progenitor cells, (F) *VCAN* for astrocytes, and (G) *CD74* for microglia.

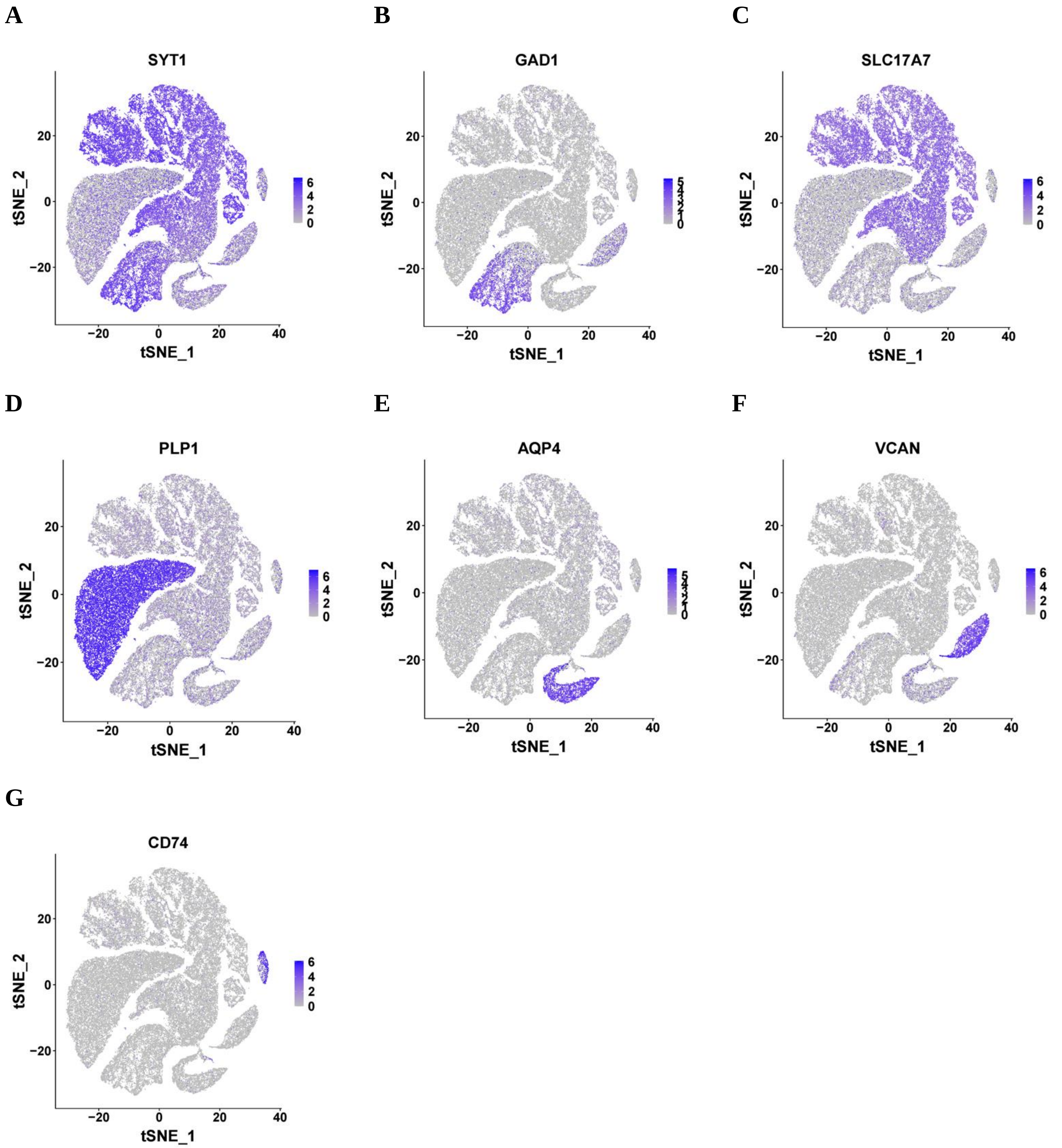

Supplementary Figure 9. Cell-type distribution of co-expressed genes in *APOE* ε2/ε3 AD cases reported in Supplemental Table S 13.

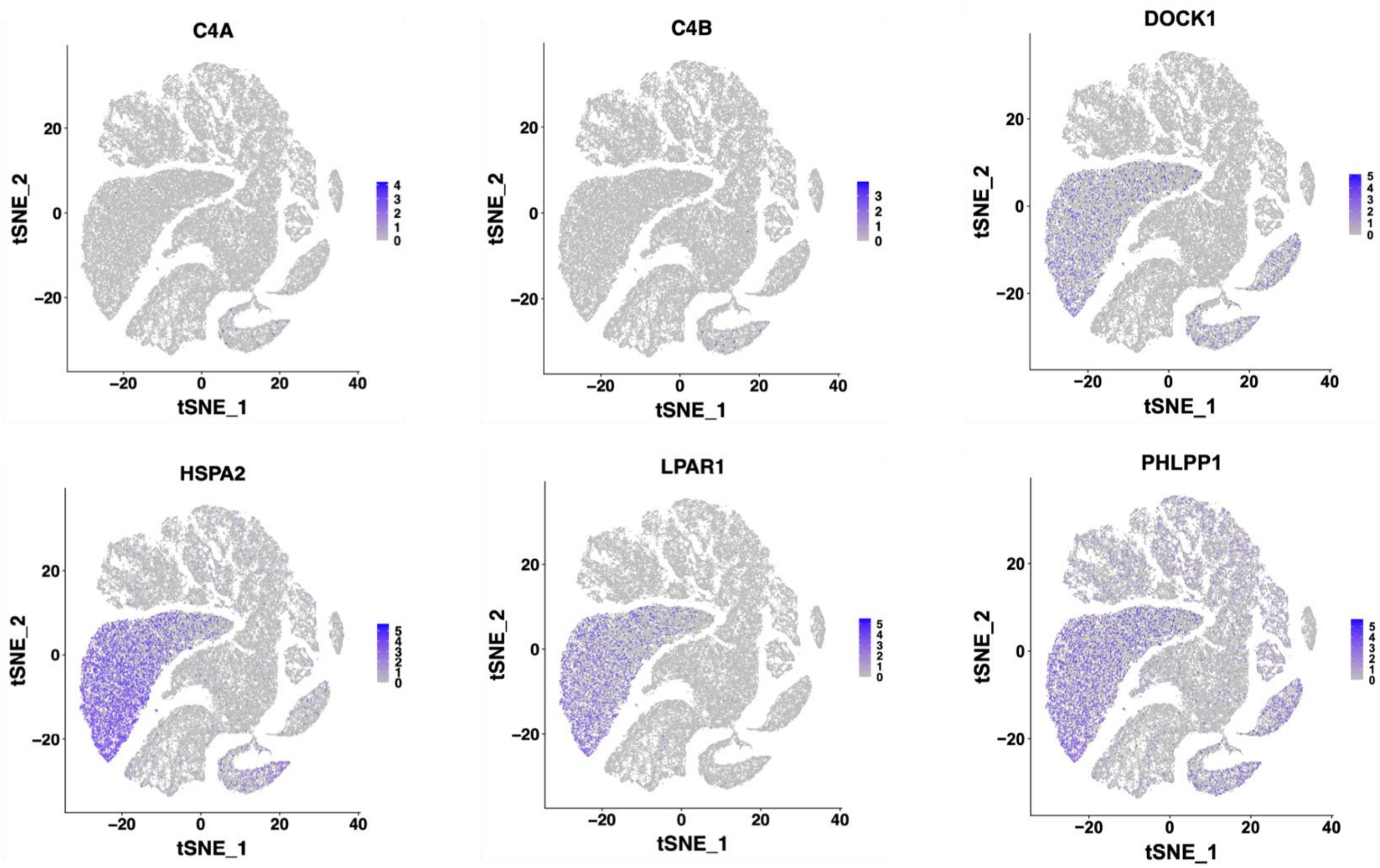

Supplementary Figure 10. Single cell expression of (A) *C4A* and (B) *C4B* in *mus musculus*. Results shown were derived from data obtained from Zhang Y et al. (J Neurosci. 2014; 34:11929–11947).

(A)

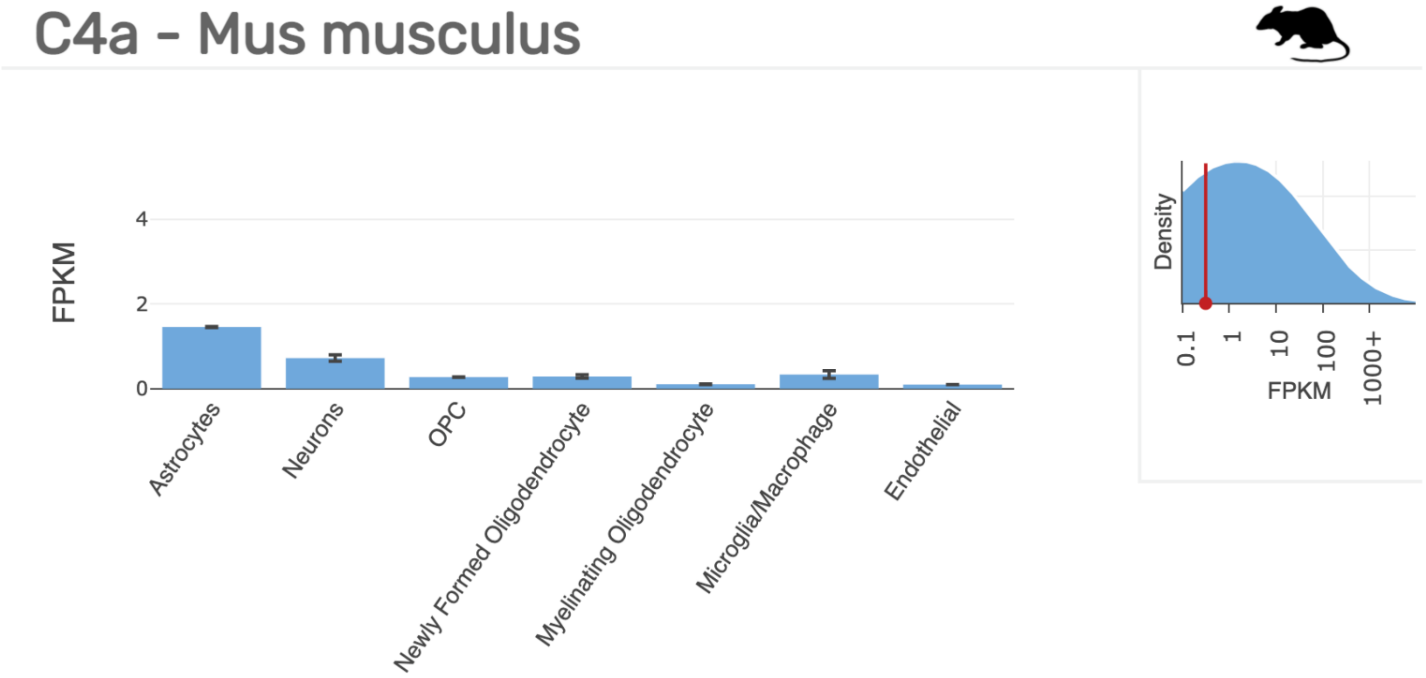

(B)

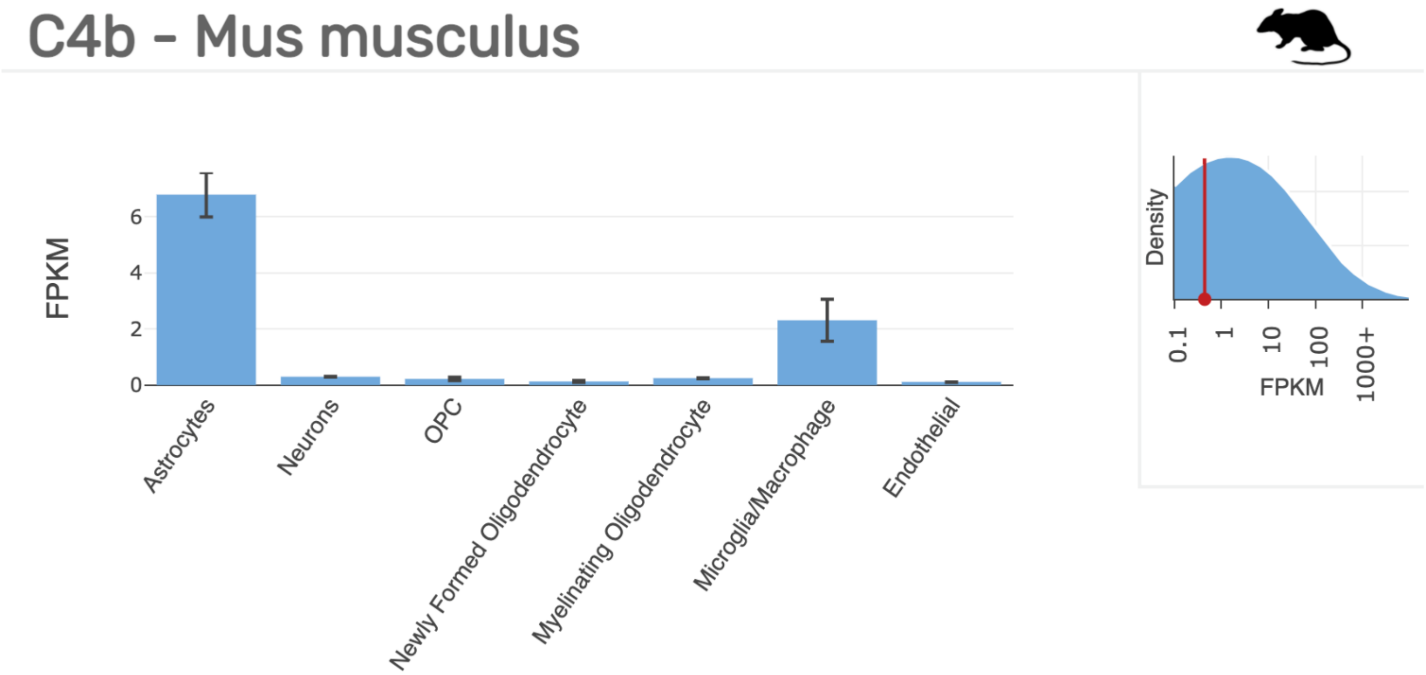

Supplement: Supplementary file 1 — Supplemental Material [file 41380_2021_1266_MOESM1_ESM.pdf]
